# Complete Genome Sequence of a Colistin-Resistant Uropathogenic Escherichia coli Sequence Type 131 *fimH*22 Strain Harboring *mcr-1* on an IncHI2 Plasmid, Isolated in Riyadh, Saudi Arabia

**DOI:** 10.1128/MRA.00104-19

**Published:** 2019-05-02

**Authors:** Majed F. Alghoribi, Michel Doumith, Mathew Upton, Sameera M. Al Johani, Maha Alzayer, Neil Woodford, Matthew J. Ellington, Hanan H. Balkhy

**Affiliations:** a Infectious Diseases Research Department, King Abdullah International Medical Research Center, Riyadh, Saudi Arabia; b King Saud bin Abdulaziz University for Health Sciences, Riyadh, Saudi Arabia; c National Infection Service, Public Health England, London, United Kingdom; d Faculty of Medicine and Dentistry, University of Plymouth, Plymouth, United Kingdom; e Infection Prevention and Control Department, King Abdulaziz Medical City, Riyadh, Saudi Arabia; f Department of Pathology and Laboratory Medicine, King Abdulaziz Medical City, Riyadh, Saudi Arabia; University of Delaware

## Abstract

We report the complete genome sequence of a colistin-resistant strain of uropathogenic Escherichia coli, isolated in January 2013 at King Abdulaziz Medical City (KAMC), Riyadh, Saudi Arabia. The isolate (named SA186) was sequence type 131 (ST131) and belonged to serotype O25b-H4 and clade B (*fimH*22).

## ANNOUNCEMENT

In response to the emergence of the plasmid-mediated colistin resistance gene *mcr-1*, first reported in China (1), retrospective PCR screening of a surveillance collection of uropathogenic Escherichia coli (UPEC) isolates from King Abdulaziz Medical City (KAMC) in Saudi Arabia identified a positive isolate belonging to the globally disseminated sequence type 131 (ST131) clone (2). ST131 is an established high-risk clone causing community-acquired and hospital-acquired urinary tract and bloodstream infections and is a major public health concern (3, 4).

We determined the complete genome sequence of the *mcr-1*-positive ST131-O25:H4-*fimH*22 clade UPEC isolate SA186, which was isolated from a 2-year-old female patient with urinary tract infection in January 2013. Susceptibility testing performed using the Vitek II XL system (bioMérieux, France) showed that SA186 was resistant to ampicillin (MIC, ≥32 mg/liter), piperacillin (MIC, ≥128 mg/liter), gentamicin (MIC, ≥16 mg/liter), tobramycin (MIC, 8 mg/liter), norfloxacin (MIC, 2 mg/liter), and trimethoprim (MIC, ≥320 mg/liter) but susceptible to third-generation cephalosporins and carbapenems. SA186 resistance to colistin (MIC, 8 mg/liter) was confirmed by the microbroth dilution method in accordance with the Clinical and Laboratory Standards Institute (CLSI) (5).

The isolate was grown on Columbia agar (Oxoid) at 37°C, and genomic DNA was extracted from an overnight single starting colony culture using the QIAsymphony SP instrument following the manufacturer’s instructions (Qiagen). Genomic DNA (>50 kb) was sheared by passage through a 26-gauge needle, and the size of the fragments thus generated (>30 kb) was checked on a fragment analyzer. Fragmented DNA cleaned with Ampure XP beads (Agencourt) was used for library preparation using the SMRTbell template prep kit version 1.0 (PacBio) according to the manufacturer’s instructions. The complete genome sequence of SA186 was determined using the PacBio RS II single-molecule real-time (SMRT) instrument at the Centre for Genomic Research at the University of Liverpool (United Kingdom).

PacBio sequence reads were assembled following the Hierarchical Genome Assembly Process (HGAP) workflow version 2.3.0 with the default settings (6). Briefly, high-quality reads were first filtered and corrected by mapping single-pass reads to the longest reads to generate highly accurate preassembled reads which were later assembled into contigs using Celera Assembler version 8.1 and were further polished with Quiver. Overlapping sequences at the ends were trimmed manually using CLC Main Workbench version 6.9.1 (Qiagen Bioinformatics) to circularize the assembled contigs. The accuracy of the contigs thus generated was confirmed with the assembly generated by Canu software version 1.0 (7). The assembly generated six contigs using a total of 78,196 reads (number of bases, 1,287,850,329; mean read length, 16,469 bp) with an average sequencing coverage depth of 185.73×. The NCBI Prokaryotic Genome Annotation Pipeline was used for coding sequence detection and annotation (8).

The genome of SA186 consisted of one 4,828,837-bp chromosome with a GC content of 50.68% and five plasmids designated pSA186-MCR-1 (241,600 bp, 46.24% GC content), pSA186-2 (198,748 bp, 49.36% GC content), pSA186-3 (113,162 bp, 50.66% GC content), pSA186-4 (96,658 bp, 48.11% GC content), and pSA186-5 (106.936 bp, 48.88% GC content). Plasmid pSA186-MCR-1 carried the *mcr-1* gene and belonged to the incompatibility group IncHI2 and plasmid multilocus sequence typing (pMLST) subtype ST4 (9). Identification of antibiotic resistance genes was performed using ResFinder version 2.1 (10) and the Comprehensive Antimicrobial Resistance Database (CARD) (11). It coharbored genes encoding resistance to aminoglycosides (*aadA1*, *aadA2*, *aphA1*, *strA*, *strB*), penicillins (*bla*_TEM-1B_), macrolides (*mphA*, *mefB*), chloramphenicol (*cml*, *floR*), streptothricin (*sat1*), sulfonamides (*sul3*), tetracyclines [*tet*(A)], and trimethoprim (*dfrA14*) and shared 99% nucleotide identity with IncHI2 plasmid pSA26-MCR-1 (GenBank accession no. KU743384), which was previously described in a strain of E. coli (ST68) isolated from a patient in Saudi Arabia in June 2012 (12). Plasmid pSA186-MCR-1 was also highly similar (>90% nucleotide identity) to *mcr-1*-positive plasmids pS38 (GenBank accession no. KX129782) (13), p14408_M1 (GenBank accession no. LT599829) (14), and pHNSHP45-2 (GenBank accession no. KP347127) (1), which were recovered from human and animal E. coli isolates belonging to ST602 (Switzerland), ST362 (Germany), and an undetermined ST (China), respectively.

In summary, this is the first report of a complete genome sequence of a multiresistant ST131 *fimH*22 subclone non-extended-spectrum β-lactamase (non-ESBL)/carbapenemase-producing UPEC strain from Saudi Arabia, which harbors the *mcr-1* gene. Its detection highlights a new challenge in the emergence and evolution of antimicrobial resistance plasmids in the Arabian Peninsula.

### Data availability.

The complete genome sequence of UPEC isolate SA186 has been deposited in GenBank under the accession no. CP022730 for the chromosome, CP022731 for pSA186_2, CP022732 for pSA186_3, CP022733 for pSA186_4, CP022734 for pSA186_5, and CP022735 for pSA186_MCR. These sequences are part of BioProject no. PRJNA395653, and the SRA accession number is SRX3097879.
